# Overexpression of p75^NTR^ in Human Seminoma: A New Biomarker?

**DOI:** 10.3390/life11070629

**Published:** 2021-06-29

**Authors:** Anna Perri, Vittoria Rago, Rocco Malivindi, Lorenza Maltese, Danilo Lofaro, Emanuela Alessandra Greco, Luigi Tucci, Renzo Bonofiglio, Margherita Vergine, Sandro La Vignera, Eusebio Chiefari, Antonio Brunetti, Antonio Aversa

**Affiliations:** 1 Kidney and Transplantation Research Center, Annunziata Hospital, 89100 Cosenza, Italy; anna.perri@unical.it (A.P.); danilo.lofaro@unical.it (D.L.); r.bonofiglio@aocs.it (R.B.); 2 Department of Pharmacy, Health and Nutritional Sciences, University of Calabria, 87036 Rende, Italy; rocco.malivindi@unical.it; 3 Department of Pathological Anatomy, “Pugliese-Ciaccio” Hospital, 88100 Catanzaro, Italy; lorenza.maltese@alice.it (L.M.); gigitucci@libero.it (L.T.); 4 deHealth Lab—DIMEG, UNICAL, Arcavacata di Rende (C.S.), 87036 Cosenza, Italy; 5 Department of Health Sciences, University “Magna Græcia”, 88100 Catanzaro, Italy; emanuela.greco@unicz.it (E.A.G.); chiefari@unicz.it (E.C.); brunetti@unicz.it (A.B.); 6 Department of Experimental and Clinical Medicine, University “Magna Græcia”, 88100 Catanzaro, Italy; margherita.vergine@unicz.it (M.V.); aversa@unicz.it (A.A.); 7 Department of Clinical and Experimental Medicine, University of Catania, 95100 Catania, Italy; sandrolavignera@unict.it

**Keywords:** testicular germ cell tumors (TGCTs), human seminoma, p75 neurotrophin receptor (p75^NTR^), p75^NTR^-signaling

## Abstract

Several studies have demonstrated that the p75^NTR^ low-affinity receptor of Nerve Growth Factor (NGF), is produced in abnormally large amounts in several human cancer types. However, the role of p75^NTR^ varies substantially depending on cell context, so that a dual role of this receptor protein in tumor cell survival and invasion, as well as cell death, has been supported in recent studies. Herein we explored for the first time the expression of p75^NTR^ in human specimens (nr = 40) from testicular germ cell tumors (TGCTs), mostly seminomas. Nuclear overexpression of p75^NTR^ was detected by immunohistochemistry in seminoma tissue as compared to normal tissue, whereas neither NGF nor its high-affinity TrkA receptor was detected. An increased nuclear staining of phospho-JNK, belonging to the p75^NTR^ signaling pathway and its pro-apoptotic target gene, p53, was concomitantly observed. Interestingly, our analysis revealed that decreased expression frequency of p75^NTR^, p-JNK and p53 was related to staging progression, thus suggesting that p75^NTR^ may represent a specific marker for seminoma and staging in TGCTs.

## 1. Introduction

Testicular germ cell tumors (TGCTs) are the most common tumors in adolescents and young men, accounting for almost all testicular cancers and their incidence is rising especially among Caucasians [1]. TGCTs are divided into seminoma germ cell tumors (SGCT) and non-seminoma germ cell tumors (NSGCT), the latter including undifferentiated (embryonal carcinoma) or differentiated (teratoma, yolk sac tumor and choriocarcinoma) tumors [2]. TGCTs have exceptional cure rates compared to other tumors, as 95% of affected men survive over 5 years [3]. Therefore, accurate staging and correct histopathological recognition of these tumors are critical for targeted therapy and optimal outcomes. Recent proteomic analyses of TGCTs identified novel proteins that might be used to better understand the molecular mechanism(s) involved in TGCTs [4]. As reported elsewhere [5], the histogenesis of TGCTs is complex and it is thought that they might develop from premalignant intratubular germ cell neoplasia (IGCN) that progresses toward invasive seminoma and/or non-seminoma after puberty when cells start to proliferate under the influence of hormones [6]. It is believed that TGCTs may arise from the failure of normal maturation of gonocytes, although, to date, the exact molecular derangements underlying this transformation are not clearly understood, even if the most common genetic finding is the gain of genetic material from chromosome 12p [7]. Since mammalian spermatogenesis is an intricate sequential process of germ cell differentiation from primordial germ cells or spermatogonial stem cells to functional haploid sperm that occurs via a complex interaction between germ and somatic cells, a better knowledge of the regulatory control of spermatogenesis should provide crucial insights into the occurrence and features of TGCTs [8,9,10,11]. Studies in human and animal models have demonstrated that during testicular development, the neurotrophins, a family of polypeptide growth factors and their receptors, are expressed in germ cells throughout their development and also in somatic cells, suggesting that the activation of their receptors may be important in testicular development and spermatogenesis [12,13,14,15].

Among neurotrophins, Nerve Growth Factor (NGF) and its high-affinity receptor, TrkA, exerts before birth an important role in seminiferous cord formation, germ cell differentiation and Sertoli cell viability; after birth, they are involved in sperm motility and acrosome reaction. Fewer data are available on the potential function of the low-affinity NGF receptor p75^NTR^, a member of the tumor necrosis receptor superfamily, which seems to have important implications in early testicular development [16]. Interestingly, immunohistochemistry studies have demonstrated that NGF/TrkA/p75^NTR^ are expressed in human breast, ovarian and prostatic cancers, suggesting that they may represent new diagnostic markers. Concomitantly, in vitro and in vivo cancer models showed that NGF is involved in cancer cell progression, invasion and chemoresistance, underlying that the NGF axis may represent a new therapeutic target in these tumors [17].

To our knowledge, there are no studies that have investigated the expression pattern of NGF/TrkA and p75^NTR^ in testicular cancer. To better understand the potential implications relevant for the NGF signaling pathway in TGCTs, the objective of the current study was to explore the expression of NGF and its receptors particularly in human testicular seminoma, the most common histological type of TGCTs.

## 2. Materials and Methods

### 2.1. Antibodies

The following primary antibodies were used: anti-p75^NTR^, anti-NGF, anti-TrkA, anti-p53 (Santa Cruz Biotechnology, Santa Cruz, CA, USA), anti-phospho-JNK; (Cell Signaling Technology, Milan, Italy).

Biotinylated goat-anti-rabbit, anti-mouse, anti-donkey IgGs were used as secondary antibodies (Santa Cruz Biotechnology, Santa Cruz, CA, USA).

### 2.2. Human Tissues

Forty formalin-fixed and paraffin-embedded samples of TGCTs, stored from the 1 January 2015 to 31 December 2019 in the archives of the Division of Pathology, Hospital “A. Pugliese”, Catanzaro (Italy), were collected. Only samples from primary tumors before chemotherapy were selected. The clinicopathological data collected included age, date of diagnosis, histological type, tumor grade (when applicable) and the presence of vascular invasion, according to testicular cancer guidelines of Associazione Italiana di Oncologia Medica (AIOM) 2018 [18]. The median age of the 40 patients was 38 years (ranging from 25 to 51 years); all patients were Caucasian. More than half of the collected specimens presented with stage I neoplasms (67.5%) and the remaining were among stages II (17.5%), III (12.5%) and IV (2.5%), respectively. Seminoma was the most frequent histological type (65%), while embryonal carcinoma and mixed GCT were less represented (27.5% and 7.5%, respectively). Control testicular tissues were obtained from 4 male patients (aged 31 and 44 years), showing testes with a like-sarcoidosis granulomatous lesion. At the time of orchidectomy, all patients gave their informed consent to use the remaining portions of tissue specimens for research purposes after their primary use for routine histologic staining. Therefore, for this study, no formal ethical approval was required for processing archival testicular tissue.

### 2.3. Tissue Microarray (TMA) Construction and Validation

All samples were analyzed, independently, by two pathological experts to confirm the diagnosis and delimitation of tumor areas for TMA cores. For each sample, both the tumor area and the corresponding normal tissues were selected (when available and sufficient) for triplicate cores of 1.0 mm. For TMA validation, 10 samples were randomly selected. For these 10 samples, the immunohistochemical analysis was performed on both TMA and whole sections and the results were compared.

### 2.4. Histopathological Analysis

A complete morphological analysis of control and seminoma samples was performed at the Division of Pathology, Hospital “A. Pugliese”, Catanzaro (Italy), upon Hematoxylin & Eosin staining. The histology slides were independently examined by the pathologists, blinded to the clinical diagnosis and the observations made by the other pathologists, careful description of tissue structural features and cellular component was performed on each sample analyzed.

### 2.5. Immunohistochemistry

The immunohistochemical experiments were carried out on paraffin-embedded sections from all samples. Sections of 5 μm thick, after heat-mediated antigen retrieval, were obtained. Immunodetection was performed at 4 °C overnight, using the specific primary antibodies anti-NGF (1:100), anti-TrkA (1:100), anti-p75^NTR^ (1:100), anti-p53 (1:100), anti-posho-JNK (1:100). Then, biotinylated IgG (1:600) was applied for 1 h at room temperature, followed by avidin-biotin complex (ABC)/horseradish peroxidase (HRP). Immunoreactivity was visualized by using diaminobenzidine chromogen (DAB). Sections were also counterstained with hematoxylin. The specificity of the Abs was verified by using normal rabbit serum and normal mouse serum, respectively, instead of the primary Abs. After immunohistochemistry analysis, slides of tumor samples were visualized using an Olympus BX41 microscope and the images were taken with CSV1.14 software, using a CAM XC-30 for image acquisition.

### 2.6. Scoring System

Immunoreactivity for human neoplastic tissues was scored using the “Allred Score” [19], which combines a proportion and an intensity score. A proportion score was assigned representing the estimated proportion of positively stained tumor cells on a scale from 0 to 5. An intensity score was assigned by the average estimated intensity of staining in positive cells on a scale from 0 to 3. Proportion score and intensity score were added to obtain a total score that ranged from 0 to 8. A minimum of 100 cells were evaluated in each slide. Six serial sections were scored for each sample.

### 2.7. Statistical Analysis

The results obtained with the human samples were analyzed using Prism GraphPad (version 9.0). ROC curve was used to define the final score cutoff for positivity, based on the area under the curve. The frequency of protein expression and comparison with clinicopathological data as well as the differences in the scores between seminoma and control samples were analyzed using the one-way ANOVA. The Wilcoxon test was used after ANOVA as post hoc test. The agreement between TMA and the whole section was evaluated by the accuracy of the method.

## 3. Results

### 3.1. Morphology Analysis in Control Testis and Seminoma

**Figure 1:**
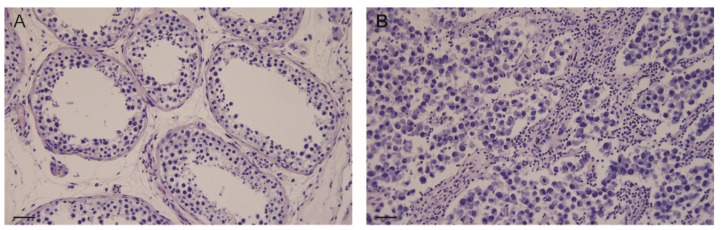
Hematoxylin and Eosin staining in control testis (**A**) and seminomas (**B**). Scale bars: 25 μm.

The morphological analysis of control testes displayed seminiferous tubules with active spermatogenesis and Sertoli cells in the basal compartment, Leydig cells were observed in the interstitial tissue (Figure 1A). The same analysis in Seminoma samples showed large cells with prominent cell membranes containing clear cytoplasm and a hyperchromatic nucleus with a prominent nucleolus. The Seminoma cells are arranged in small clusters separated by connective tissue septae into sheets or cords. Frequently, there is a lymphocytic infiltrate (Figure 1B).

### 3.2. Immunohistochemical Localization of p75^NTR^, NGF and TrkA in Control Testis and TGCC

**Figure 2:**
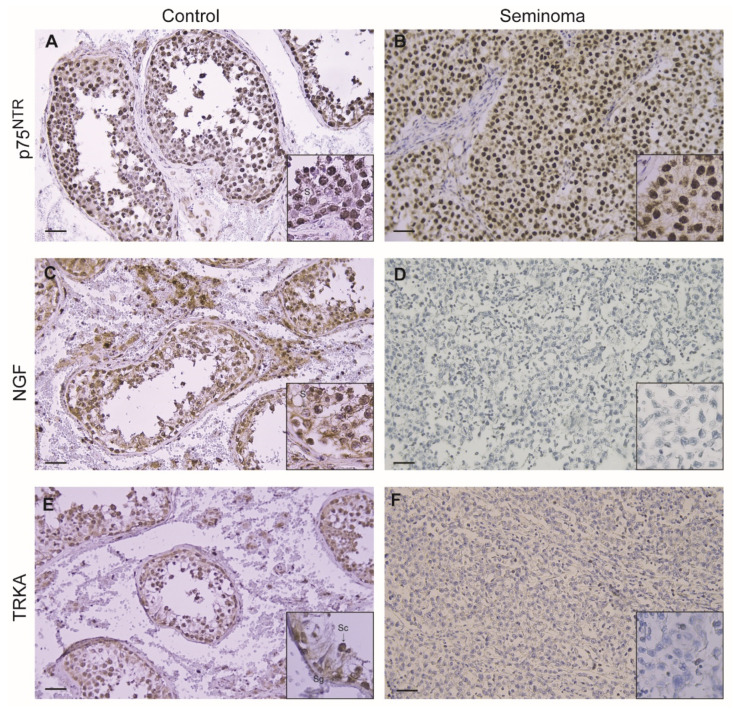
Immunohistochemical expression of p75^NTR^ (**A**,**B**), NGF (**C**,**D**) and TrkA (**E**,**F**), in control testis and seminomas. (S) Sertoli cells; (Sg) Spermatogonia; (Sc) Spermatocytes; (Sd) Spermatides. Scale bars: 25 μm. Inserts: higher magnifications of the images.

Control testis revealed an intense p75^NTR^ immunoreactivity in tubular compartments, particularly in spermatogonia, spermatocytes and spermatids, while Sertoli cells were immunonegative in the interstitium; the Leydig cells showed the same strong immunopositivity. In tumoral samples, a very strong nuclear immunoreactivity was shown in big neoplastic cells while the leukocytes were immunonegative, (Figure 2A,B; Table 1). A moderate NGF immunoreactivity was evidenced only in Sertoli cells cytoplasm and in spermatogonia as well as in Leydig cell of control testes, while tumoral samples were immunonegative (Figure 2C,D; Table 1). Furthermore, although less intense, the TrkA immunopositivity in control testes was evidenced in the same germinal cells exhibiting p75^NTR^ immunoreactivity, while tumoral samples resulted immunonegative (Figure 2E,F; Table 1). The same immunopositivity pattern for p75^NTR^ was observed only in 4/14 of non-seminoma samples; all samples were immunonegative for NGF and TrkA (data not shown).

Cutoffs for positivity of the p75^NTR^ marker were defined based on the area under the ROC curve, considering the sum of the score and the occurrence of clinical events (Table 2).

### 3.3. Immunohistochemistry of p53 in TGCC

**Figure 3:**
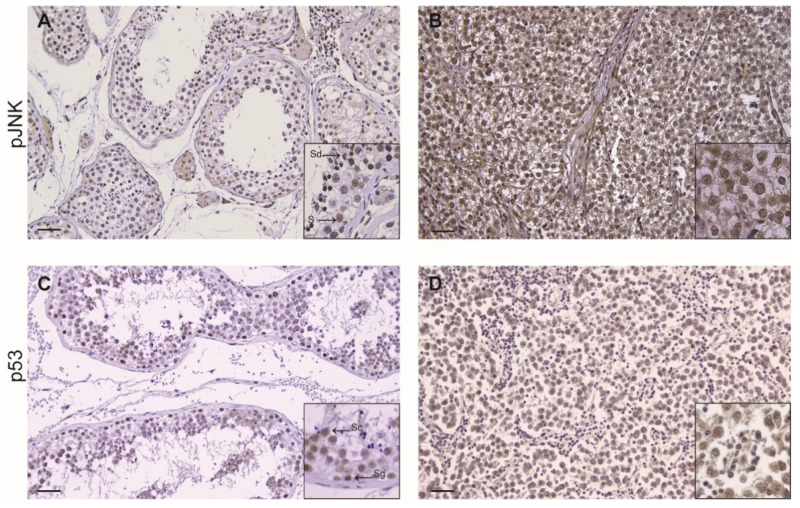
Immunohistochemical expression of pJNK (**A**,**B**) and p53 (**C**,**D**) in control testis and seminomas. (S) Sertoli cells; (Sg) Spermatogonia; (Sc) Spermatocytes; (Sd) Spermatides. Scale bars: 25 μm. Inserts: higher magnifications of the images.

Interestingly, in seminoma tissue, we evidenced an increased expression of p-JNK as compared to controls; the tumoral cells showed a strong p-JNK immunopositivity while the lymphocytes were un-stained. In the control samples the positivity was confined in Sertoli cells, in spermatids and Leydig cells, respectively (Figure 3A,B; Table 3). Moreover, immunohistochemical analysis revealed a strong nuclear immunoreactivity of p53 in seminoma samples compared to control tissue sections, where p53 immunolocalization was stronger in spermatogonia and moderate in spermatocytes (Figure 3C,D, Table 3).

### 3.4. Validation of TMA Method

Analysis of agreement between TMA and whole tissue sections showed an accuracy for the TMA method of 80% for NGF, 98% for TrkA and 78% for p75^NTR^.

### 3.5. Clinico-Pathological Significance of p75^NTR^, p-JNK and p53

**Figure 4:**
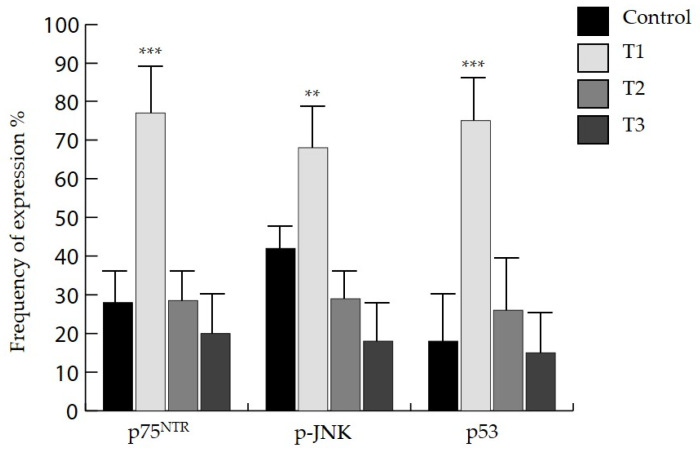
Frequency of expression of p75^NTR^, p-JNK and p53 in different tumor T stages versus control (** *p* < 0.005, *** *p* < 0.001).

To explore the potential clinico-pathological significance of NGF, TrkA and p75^NTR^, we investigated the frequency of expression of the above-reported markers according to the staging (Table 4). Moreover, our analysis revealed that when compared to control tissue samples, p75^NTR^, p-JNK and p53 showed higher expression in samples with T1 tumor stage (*p* < 0.001) (Figure 4).

## 4. Discussion

As far as we are aware, this study represents the first description of the overexpression of p75^NTR^ in TGCTs, particularly in testicular seminoma and of its downstream signaling molecules, phospho-JNK and p53, whose expression decreases as tumor staging worsens. Different studies have proven that p75^NTR^ plays opposite roles in the context of different cancers, as it acts as a tumor suppressor in carcinomas of prostate, bladder, stomach and liver [20,21,22], whereas, in melanoma, pancreatic carcinoma, glioma and breast cancer, p75^NTR^ acts as a tumor-promoting function facilitating survival and invasion of cancer cells [23,24,25].

Although the involvement of p75^NTR^ has been investigated in tumors affecting the female reproductive system, to our knowledge there are no studies that have explored the expression of p75^NTR^ in TGCTs, in particular in testicular seminoma, which represents the most frequent testicular neoplasm in young men [1,26].

Previous studies have been carried out to identify new molecular markers for TGCTs. Preliminary reports demonstrated that most cells in seminoma express Pituitary-tumor-transforming-gene 1 (PTTG1), as well as Octamer-binding transcription factor 4 (OCT-4) and Krüppel-like factor 4 (KLF-4) [27]. The authors firstly demonstrated that PTTG1 marks some specific OCT4- and KLF4-positive tumor cells, mainly localized at the periphery of the neoplasm. In the intratubular infiltration areas, nests of cells expressing both OCT4/KLF4 and PTTG1 presence were consistent for a sub-population of tumor stem cells OCT4- and KLF4- positive [28]. In the present study, we aimed to investigate a new molecular pathway and found that the overexpression of p75^NTR^ may have a pathogenetic role in testicular germ cell cancer development, which may occur in an NGF-independent manner [29,30], as confirmed by immunohistochemical Allred-score of negative NGF staining and its pro-survival receptor, TrkA, in tumoral tissue. Interestingly, Micera et al. reported that the biological effect mediated by the NGF axis in both normal and cancer cells is related to the TrkA/p75^NTR^ ratio and that the p75^NTR^ expression might facilitate cell proliferation in the absence of TrkA [31].

We can speculate that p75^NTR^ may be a biomarker of the transformed primordial germ cells, which represent the characteristic cellular pattern of testicular seminoma, in particular its pure forms [32]. The pathogenesis of overall TGTCs remains unexplored, although it is known that they originate from transformed gonocytes or undifferentiated spermatogonia [33].

Different observations using several malignancies refer to cells with a remarkable self-renewal potential and extensive proliferation capacity [34,35,36], expressing markers that characterize the stem cells of the original normal tissue [37] and that are strongly involved in growth and tumor propagation. A growing body of evidence has identified p75^NTR^ as a robust cell surface biomarker for neural cancer-initiating or stem-like cells [38]. Interestingly, Okumura et al. demonstrated that in the stem/progenitor cell fraction of normal esophageal epithelial cells, p75^NTR^ is necessary for tumor survival and maintenance [39]. Moreover, the in vivo study of Boiko et al. reported that melanoma tumor stem cells p75^NTR^-positive, but not p75^NTR^-negative, were remarkably capable of generating tumors, promoting metastasis and maintaining self-renewal [40].

Furthermore, as expected, we observed that control testicular tissue expressed phospho-JNK; as it has been reported that JNK is involved in regulating various testicular functions, like germ cell development and acrosome reactions, by controlling the expression of genes involved in the apoptosis or survival signaling pathways [41,42]. Concomitantly, we found that the overexpression of p75^NTR^ in tumor tissue was accompanied by an increased nuclear expression of its downstream signaling, phospho-JNK. This result requires further investigation, as it has been extensively demonstrated that both p75^NTR^ and JNK pathway activation can promote different biological effects, depending on cancer type [43]. Expression of p75^NTR^ has been associated with apoptosis, through activation of JNK, which controls the expression of p53 tumor suppressor and with cell survival, through the activation of nuclear factor-kB and AKT [44]. In addition to p75^NTR^ and phospho-JNK overexpression, our analysis revealed a strong nuclear immunoreactivity of p53 in the tumor sections compared to control, highlighting that hyperactivation of p75^NTR^ signaling in seminoma cells may promote cancer cell apoptosis. Furthermore, we hypothesized that p75^NTR^ overexpression may represent a positive prognostic factor in seminoma, as we observed that p75^NTR^, phospho-JNK and p53 expression frequency decreases with staging progression. These results are in agreement with previous reports, demonstrating an inverse association of p75^NTR^ expression with the neoplastic progression of prostate cancer [45,46]. Interestingly, we observed a strong p75^NTR^ immunolocalization in four non-seminoma samples (two embryonal carcinoma and two mixed TGCT); however, the small sample is not sufficient to assume a potential role of p75NTR in non-seminoma cancer.

Our study has some limitations. First, this is a retrospective study based on an archive of collected samples coming from testicular neoplasms and, therefore, the lack of access to patients’ clinical information limited the evaluation of any relationship between immunohistochemistry findings and tumor aggressiveness. In addition, we used the area under the ROC curve to assess the cutoffs for p75^NTR^ positivity and to investigate the definition of scores better associated with clinical events. Although it may vary depending on tumor type, we used this approach to estimate the possible clinical significance of p75^NTR^ as a biomarker. TMA method demonstrated accuracy for all proteins analyzed. The tumor heterogeneity, quite common in TGCTs, could be a limitation of this method. However, in this study, scores in triplicate for each histological subtype were used, in the attempt to minimize this limitation. Lastly, the present study was not aimed to clarify any molecular pathway that could yield to any relevant translational mechanism(s) linked to tumor progression.

## 5. Conclusions

In conclusion, our results suggest that p75^NTR^ may exert some role in TGCTs since the loss of its expression may represent a specific hallmark of seminoma and possible staging in TGCTs. However, the mechanism(s) underlying the controversial and paradoxical functions of p75^NTR^ in different cancer cells are not entirely explained at present. Larger translational studies are warranted to better clarify the underlying molecular mechanism by which P75^NTR^ signaling may be involved in testicular germ cell tumorigenesis and represent a new marker of better staging. Therefore, further clinical studies are needed to confirm the relevance of our findings.

## Figures and Tables

**Table 1:** Immunostaining scores (Allred score median) of p75^NTR^ NGF and TrkA in human control testes and seminoma samples.

	Control (n = 4)	Seminoma (n = 26)
p75^NTR^	4 (3–4)	8 (7–8) ***
NGF	2 (2–3)	0 (0–0)
TrkA	3 (2–4)	0 (0–0)

Immunostained scores: Total score = Proposition score + Intensity score (range 0–8). *** Seminoma *versus* Control = *p* < 0.001 (one-way Anova test).

**Table 2:** p75^NTR^ cutoff based on the area under the ROC curve.

Marker	Cutoff	Sensitivity (%)	Specificity (%)	PPV	NPV	AUC (95% CI)
p75^NTR^	≥7	67.8%	59.0%	21.4%	76.0%	0.80 (0.75–0.98)

AUC—Area under ROC curve; CI—Confidence interval; PPV—Positive predictive value; NPV—Negative predictive value.

**Table 3:** Immunostaining scores (Allred score median) p-JNK and p53 in human control and seminoma samples.

Marker	Control (n = 4)	Seminoma (n = 26)
p-JNK	3 (2–3)	7 (6–8) ***
P53	3 (2–3)	7 (6–8) ***

Immunostained scores: Total score = Proposition score+ Intensity score (range 0–8). *** Seminoma versus Control = *p* < 0.001 (one-way ANOVA test).

**Table 4:** Association of NGF, TrkA and p75^NTR^ expression with clinico-pathological parameters.

	nr	NGF		TrkA		p75^NTR^	
		Positive (%)	*p*	Positive (%)	*p*	Positive (%)	*p*
**T stage**			0.91		0.40		0.001
T1	27	1 (0.03)		5 (5.9)		21 (77.7)	
T2	7	0 (0.0)		0 (0.0)		2 (28.5)	
T3	5	0 (0.0)		0 (0.0)		1 (20.0)	
T4	1	0 (0.0)		0 (0.0)		0 (0.0)	
**Vascular invasion**			ns		ns		0.59
No	34	0 (0.0)		0 (0.0)		13 (38.2)	
Yes	6	0 (0.0)		0 (0.0)		3 (64.3)	
**Histology**			ns		ns		0.0001
Seminoma	26	0 (0.0)		0 (0.0)		25 (96.1)	
Non-seminomatous	14	0 (0.0)		0 (0.0)		4 (28.5)	

## Data Availability

Not applicable.
